# Evaluation of cancer outcome assessment using MRI: A review of deep-learning methods

**DOI:** 10.1259/bjro.20210072

**Published:** 2022-06-22

**Authors:** Yousef Mazaheri, Sunitha B. Thakur, Almir GV Bitencourt, Roberto Lo Gullo, Andreas M. Hötker, David D B Bates, Oguz Akin

**Affiliations:** ^1^ Department of Medical Physics, Memorial Sloan Kettering Cancer Center, New York, United States; ^2^ Department of Radiology, Memorial Sloan Kettering Cancer Center, New York, United States; ^3^ Department of Imaging, A.C.Camargo Cancer Center, São Paulo, Brazil; ^4^ Dasa, Sao Paulo, SP, Brazil; ^5^ Institute of Diagnostic and Interventional Radiology, University Hospital Zurich, Zurich, Switzerland

## Abstract

Accurate evaluation of tumor response to treatment is critical to allow personalized treatment regimens according to the predicted response and to support clinical trials investigating new therapeutic agents by providing them with an accurate response indicator. Recent advances in medical imaging, computer hardware, and machine-learning algorithms have resulted in the increased use of these tools in the field of medicine as a whole and specifically in cancer imaging for detection and characterization of malignant lesions, prognosis, and assessment of treatment response. Among the currently available imaging techniques, magnetic resonance imaging (MRI) plays an important role in the evaluation of treatment assessment of many cancers, given its superior soft-tissue contrast and its ability to allow multiplanar imaging and functional evaluation. In recent years, deep learning (DL) has become an active area of research, paving the way for computer-assisted clinical and radiological decision support. DL can uncover associations between imaging features that cannot be visually identified by the naked eye and pertinent clinical outcomes. The aim of this review is to highlight the use of DL in the evaluation of tumor response assessed on MRI. In this review, we will first provide an overview of common DL architectures used in medical imaging research in general. Then, we will review the studies to date that have applied DL to magnetic resonance imaging for the task of treatment response assessment. Finally, we will discuss the challenges and opportunities of using DL within the clinical workflow.

## Introduction

In recent years, there has been a dramatic increase in research studies applying artificial intelligence (AI) approaches to a wide range of decision-making problems. In cancer imaging research, deep learning (DL) has shown promising performance in a wide range of tasks, including cancer screening, tumor characterization (which includes subtype classification), treatment response, and survival outcome assessment.

With respect to treatment response assessment, imaging has played an important role in this task for decades. The first attempt to establish standardized criteria for image-based treatment response assessment dates back to the 1970s, when the International Union Against Cancer and the World Health Organization (WHO) developed an evaluation scheme to classify treatment response of solid tumors based on bidimensional measurements of tumor size in the axial plane on imaging studies.^1^ Since then, four categories have been used to classify image-based treatment response: complete response (CR), partial response (PR), stable disease (SD), and progressive disease (PD). In 2000,^2^ the Response Evaluation Criteria in Solid Tumors (RECIST) was published, providing guidance on treatment response classification based mainly on changes in tumor size. These criteria recommended unidimensional instead of bidimensional measurements to quantify tumor burden and have since become the most used criteria for estimating solid tumor burden and determining clinical treatment response. However, RECIST is known to have certain intractable limitations, particularly when it comes to precision medicine approaches to cancer. For example, RECIST criteria are limited by large inter- and intraobserver variations, especially in tumors with irregular and complex shapes. Furthermore, when tumors are treated with targeted chemotherapy or immunotherapy, assessing tumor response based on changes in tumor size is likely inadequate. In such cases, tumor response would be better reflected by morphologic, functional, and metabolic changes, such as residual cancer cell amount, extent of necrosis and fibrosis, or cystic degeneration. An alternative response criterion—the Choi criteria—was proposed for computed tomographic (CT) imaging and incorporates measurements of both tumor size and density.^3^ Within the context of the Choi criteria, a patient is regarded as responding if CT images show a 10% reduction in tumor size or a 15% reduction in CT attenuation. Based on these criteria, Choi et al showed that response among patients with metastatic gastrointestinal stromal tumor showed significantly longer progression-free interval compared with nonresponses.

In 2009, RECIST 1.1,^4^ a revised version of RECIST, was published. Additional response criteria have also been developed, including modified RECIST (mRECIST) for the evaluation of hepatocellular carcinoma (HCC) response to targeted therapy,^5^ immune-related response criteria (irRC) for the assessment of response to immunotherapy,^6^ and immune-related RECIST (irRECIST), which combines characteristics of irRC and RECIST.^7^ The irRC are based on bidimensional measurements and new lesions are incorporated for the measurement of total measurable tumor burden.^6^ irRECIST, reported by Nishino et al, requires only one-dimensional measurement and confirmation by two consecutive observations to judge complete response (CR), partial response (PR), or progressive disease (PD).^7^

DL methods have evolved since basic foundations were introduced in the 1940s, with methodologies advancing tremendously over the past decade. Qualitative and quantitative measurements on magnetic resonance imaging (MRI) data offer a promising technique for the assessment of treatment and survival outcomes. MRI and the numerous imaging sequences often acquired yield valuable information that can potentially serve as biomarkers for the assessment of treatment and survival outcomes. Suitable applications for DL methodology to MRI have the potential to enhance the prognosis and mortality assessments of cancer. DL methods promise to explore the complex relationship between MRI data and cancer outcomes.

The aim of this review is to highlight the use of DL in the evaluation of tumor response and survival outcome assessed on MRI. In this review, we will first provide an overview of common DL architectures used in general medical imaging research. Then, we will review published studies that have applied DL to MRI for the task of treatment response and survival outcome assessment in different types of cancer. Finally, we will discuss the challenges and opportunities of using DL within the clinical workflow.

### Deep-learning architectures commonly used in medical imaging

**Figure 1.**
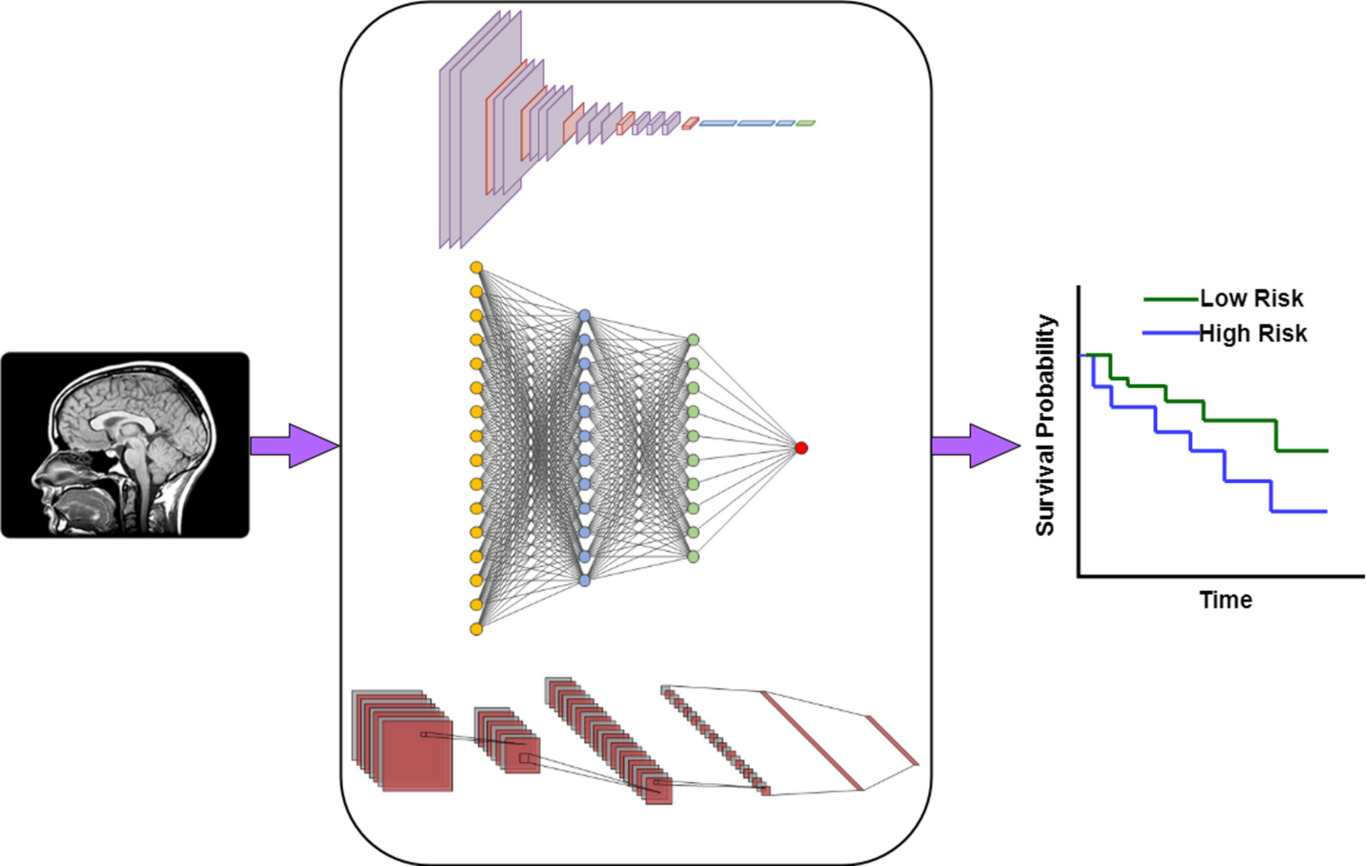
A general framework (based on deep learning algorithms) for the processing of images by classifying high and low risk of survival, thus assessing probability of treatment response. (**A**) Patient MRI images are input into a deep learning model for the purpose of training the model; (**B**) A deep-learning system developed and trained to characterize outcome assessment, such as survival probability; (**C**) The outcome of the deep-learning model is used to predict cancer outcome.

DL is a machine-learning subset where features are learned directly from raw data rather than advanced specification.^8^ DL techniques can be divided into unsupervised and supervised DL techniques. Supervised DL pre-specifies desired outputs with associated inputs while training the neural network algorithm. Accordingly, the algorithm is trained to learn relationships or transformations that allow it to predict expected outputs when given new inputs. By contrast, unsupervised DL finds relationships between variables in a given dataset without any labels. The algorithm discerns unlabeled data autonomously by relying on the extraction of inherent dominant features and patterns (Figure 1).

DL networks are characterized by hierarchical architectures consisting of multiple layers of non-linear information, whereby features in upper layers represent combinations of simpler features below. Neural networks use backpropagation as a learning algorithm to compute the gradient of the loss function for each weight in the network model. Subsequently, the gradient is used by an optimization algorithm to update model weights. In addition to calculating the gradient of a loss function with respect to variables of a model, a neural network model requires hyperparameter optimization or tuning of the learning algorithm. This task involves choosing a set of hyperparameters for a learning algorithm that yields an optimal model, or a model which minimizes a predefined loss function. Finally, cross-validation is often used to estimate the generalized performance of the model.

**Figure 2.**
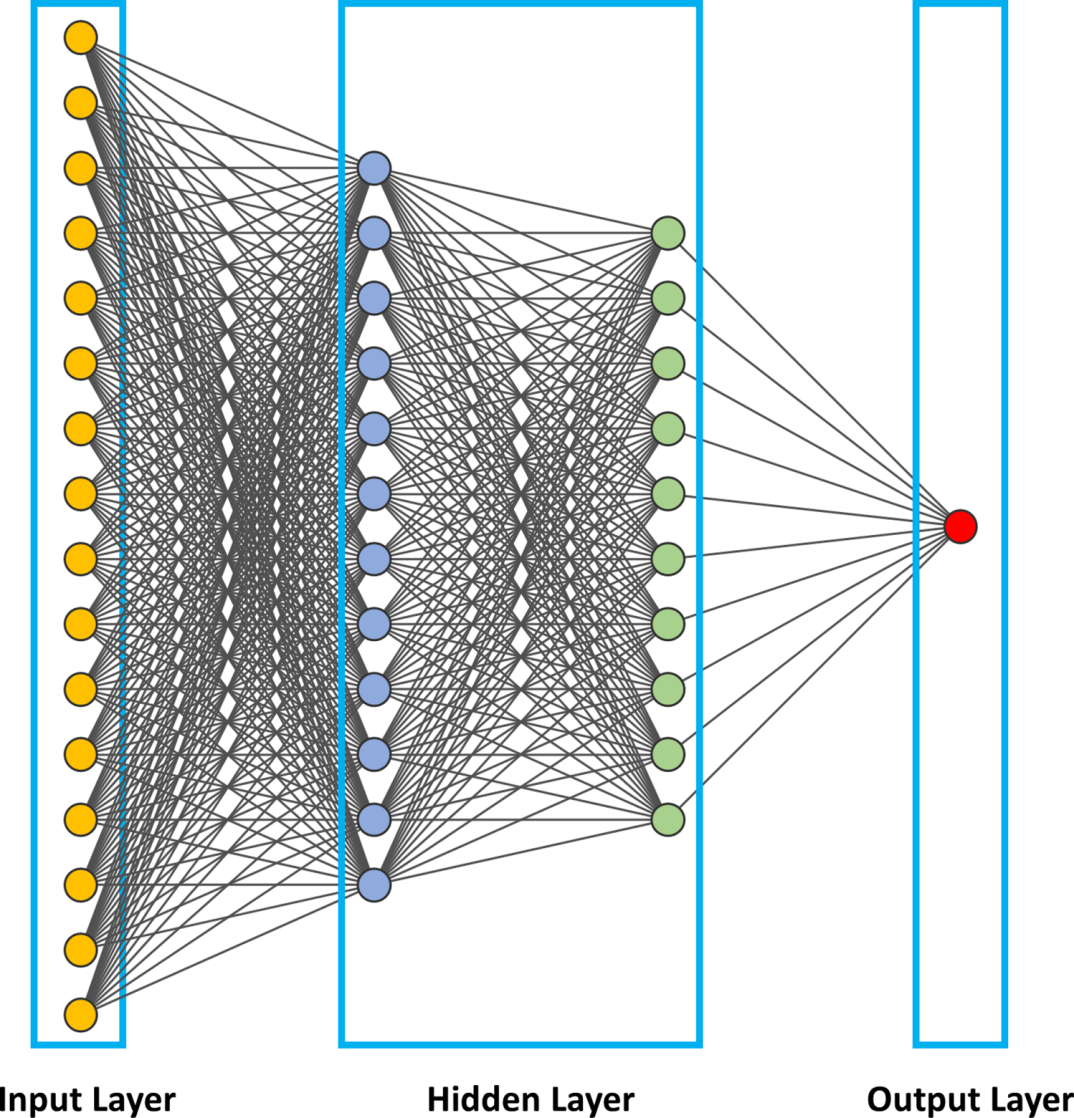
Illustration of an artificial neural networks (ANNs), the backbone of deep neural networks (DNNs). In this figure, we show a fully connected neural network where all the nodes, or neurons, in one layer are connected to the neurons in the next layer. When the input increases, fully connected networks tend to be computationally expensive, resulting in poor scalability.

We will next review DL architectures commonly used in general medical imaging research. But it is important to first consider artificial neural networks (ANNs), the backbone of deep neural networks (DNNs). ANNs are inspired by the structure and function of the human brain. ANNs can be developed based on supervised, unsupervised, or semi-supervised learning. An ANN is composed of layers of connected nodes (also called artificial neurons), configured at multiple layers (depth) and in the order of hundreds to millions (Figure 2). The objective of this configuration is to maximize the correct output as compared with a reference value. This is accomplished on each forward propagation by calculating the error and adjusting the weightings on each node. The process is repeated at each iteration (epoch), until a more accurate solution is converged.

One of the first, simplest, and most widely used ANN in practical application is the feedforward neural network (FFNN). In FFNN, information flow is always in a single and forward direction from the input nodes, through any hidden nodes, and up to the output nodes. The objective is to learn the relationship between independent variables that are network inputs and the dependent variables that are assigned as network outputs.

The construction of an ANN involves training the network on a large dataset and subsequently validating the inferences of the network on a test set. Training and optimization are achieved through a loss index measuring algorithm-associated errors. Regularization refers to strategies employed to reduce the error of the test set at the expense of increasing training error. To tune an ANN, a loss index consisting of the sum of the error and regularization term is measured, and an optimization algorithm applied to adjust the weights and bias by backpropagating the errors from the output layers in the direction of the input layers. This iterative process is repeated until the loss index is minimized or until a predetermined value is reached. A key difference between ANN and DNNs is that DNNs entail a greater number of hidden neurons, more layers, and innovative training paradigms to process larger amounts of data.

### Convolutional neural network

**Figure 3.**
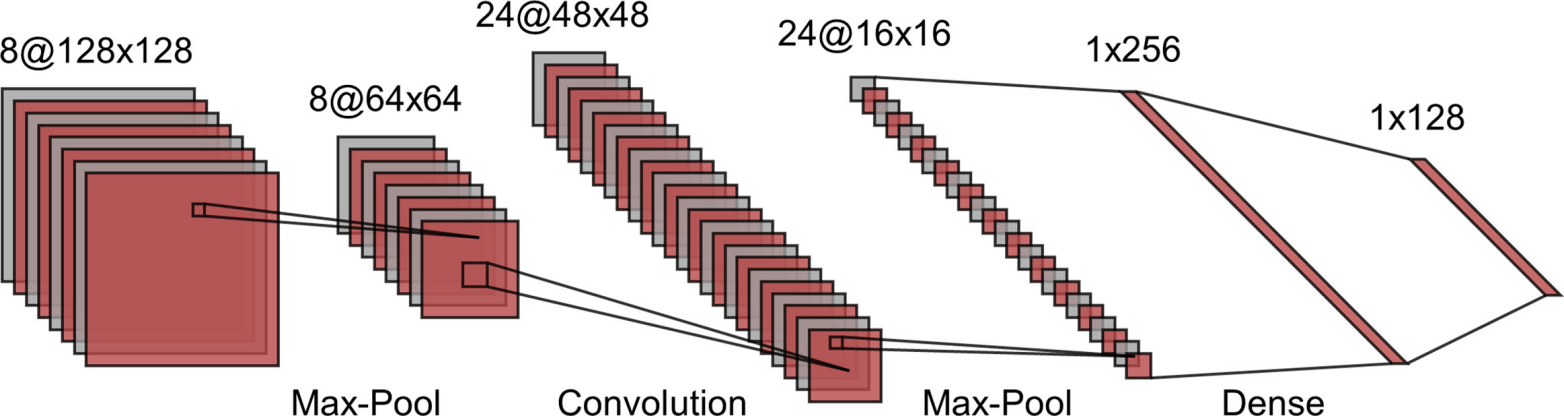
An illustration of a simple convolutional neural network including convolutional, pooling, and fully connected layers. The two-dimensional input data undergoe multiple rounds of convolution and subsample layers. Feature extraction by filters are learned through back projection. The pooling operations, including max or mean, in a region are used to reduce the number of pixels in each layer of the network. Each operation increasingly extracts higher order discriminative features. Ultimately, the output layer is a class probability based on these higher order features.

In 2012, Krizhevsky et al developed a convolutional neural network (ConvNet/CNN) that markedly improved image classification^9^ (Figure 3). CNNs are the most widely used DL architectures for medical image analysis, having been developed for tasks including image recognition, image analysis, image segmentation, video analysis, and natural language processing. The best-known CNN architectures developed to date are ZFNet,^10^ VGGNet,^11^ GoogLeNet,^12^ AlexNet,^13^ and ResNet.^14^

CNNs are multilayered neural networks with three layer types: convolutional layers, pooling layers, and fully connected layers. CNNs are designed to extract features that capture the spatial and temporal patterns of the input images. Using convolutional and pooling layers, CNNs mimic the mathematical operations of convolution and pooling. The convolution layer constitutes the essential feature of CNNs and refers to the networks’ operation based on a set of learnable filters to merge the input values and filter values onto the feature map. Pooling layers are used to reduce the dimensions of feature maps. The standard CNN employs a rectified linear unit (ReLU) as an activation function and a supplemental step to convolution. Another activation function which is very popular for neural networks is the sigmoid activation function, also called the logistic function. ReLU will give an output of zero for negative inputs but otherwise preserve the input. ReLU is the most used activation function in DL models due to its computational simplicity, representational sparsity, and linearity. As compared to the sigmoid activation function, ReLU are easier to train. The representational sparsity feature implies that the ReLU function, unlike the tanh and sigmoid activation functions, is capable of outputting a true zero value.

Further along in the network architecture, the pooling layers work to downsample the features in the convolved feature map, typically using max pooling, so that dominant features that are rotationally and positionally invariant are extracted. Finally, fully connected layers at network’s end generate the required class prediction by taking the flattened matrix from the pooling layers as input.

The main advantage of CNNs is that it captures important image features (through a backpropagation algorithm) without any human supervision. Compared with alternative network designs like FFNN, CNNs capture the spatial dependencies in an image, hence better capturing its composition. The primary disadvantages of CNNs are that they require large training data, and that they do not encode the position and orientation of the object.

**Figure 4.**
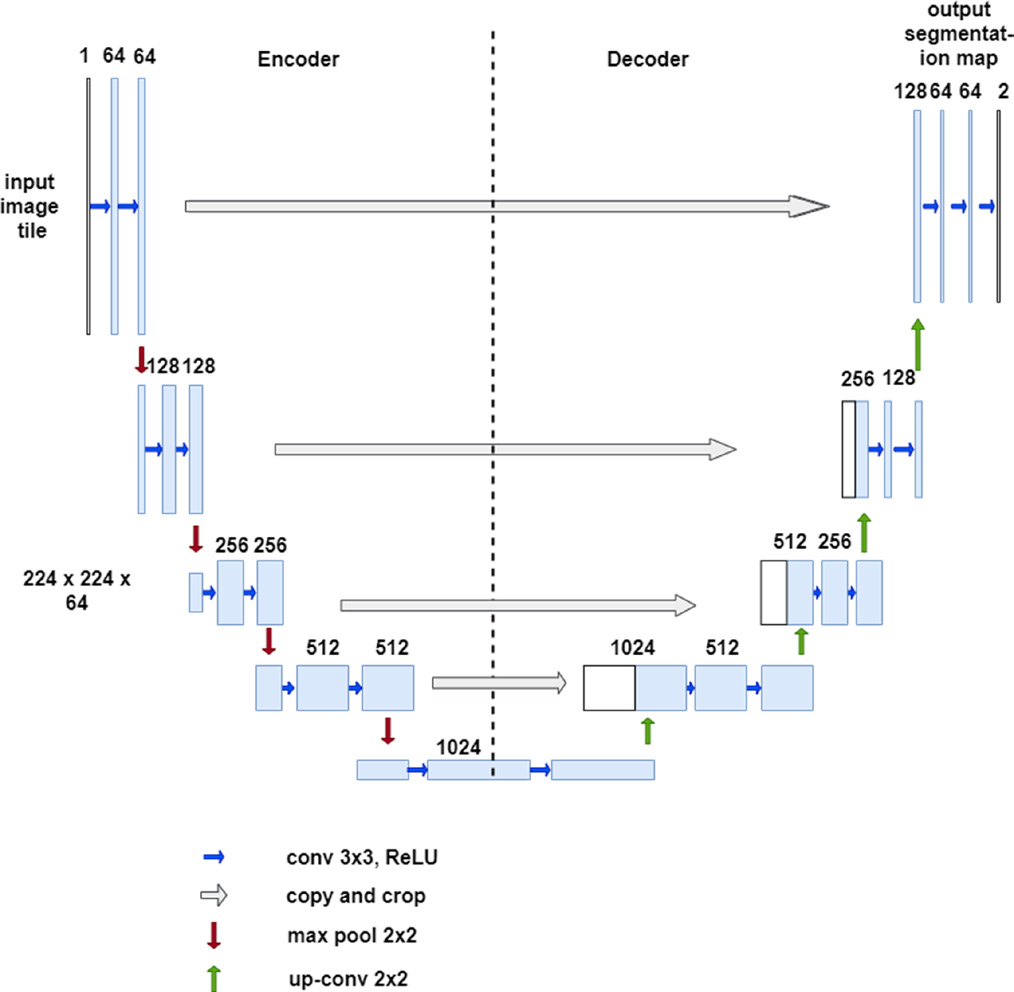
The U-net network structure has a deep-learning encoder-decoder architecture. The CNN is termed “U-net” due to the u-shaped structure. The network consists of encoder layers where there is first downsampling in the image size followed by upsampling in the expansive or decoder layer.

Many variants of the CNN architecture have been developed. For example, U-Net is a fully convolutional network developed by Ronneberger et al. in 2015 for medical image segmentation.^15^ U-Net consists of a contracting path (also known as the encoder path) that downsamples the image into a feature map, followed by an expansion path (*i.e.,* decoder path) that upsamples the feature map to the target such as the output segmentation map. During downsampling, feature information is extracted while spatial information is reduced. During upsampling, feature and spatial information are combined through a sequence of up-convolutions and concatenations, generating high-resolution features. The resultant neural network yields more precise segmentations with fewer training images. The workflow for a U-Net network is illustrated in Figure 4.

**Figure 5.**
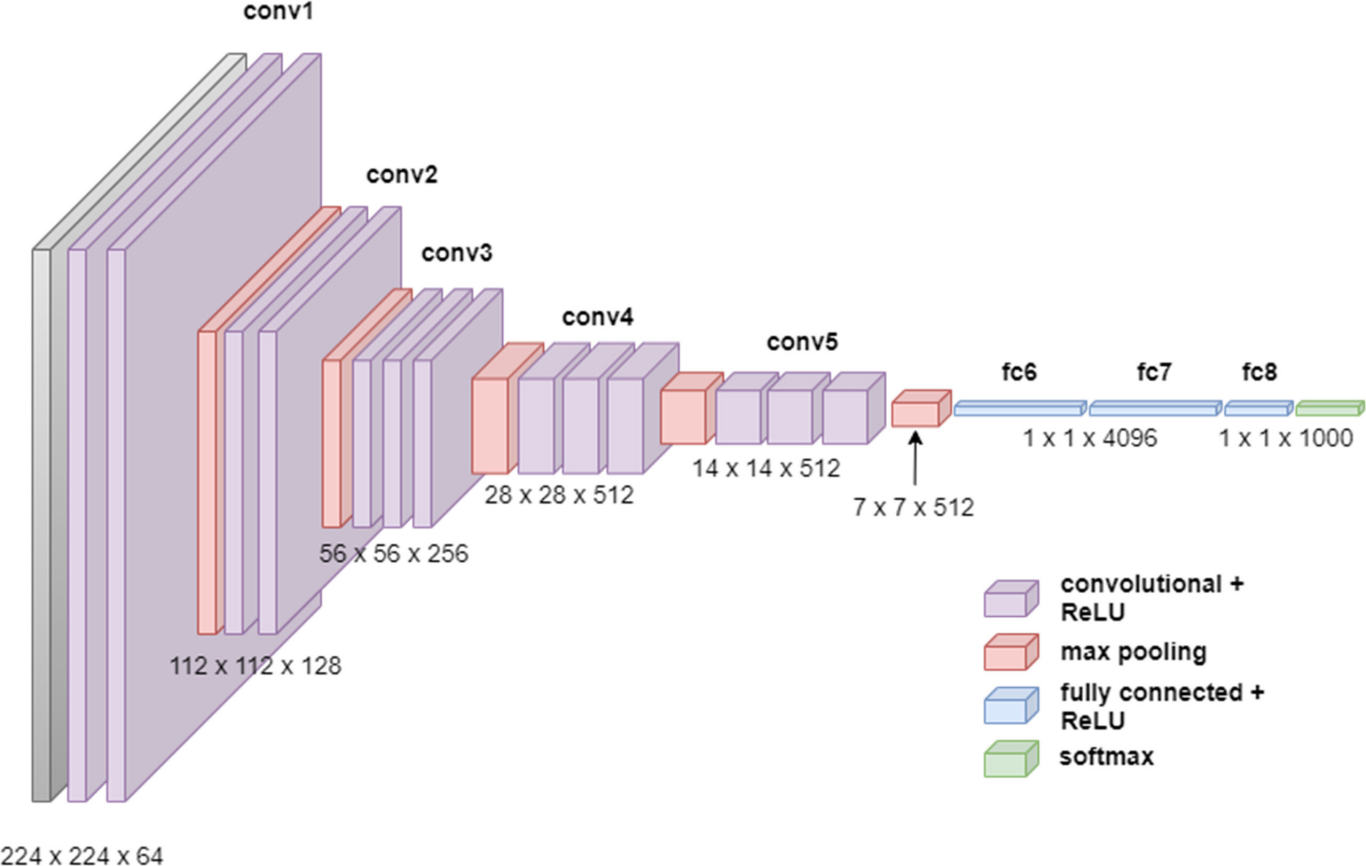
The VGG-16 architecture. The VGG16 consists of 13 convolutional layers, five max-pooling layers, and three fully connected layers. Consequently, the number of tunable parameters is 16 (13 convolutional layers and three fully connected layers).

Another CNN architecture is the VGG16, which was used to win ILSVR competition in 2014.^16^ The VGG16 network improves upon AlexNet by replacing large kernel-sized filters with multiple 3 × 3 kernel-sized filters. The network applies the same kernel size of 3 × 3 filter throughout the feature extraction part and always uses the same padding and max pool layer of 2 × 2 filter of stride 2. This arrangement of convolution and max pool layers is consistently followed throughout the network (Figure 5).

### Transfer learning

Often, training an entire network from scratch is impractical since this requires large training datasets. Should a large training dataset be unavailable, transfer learning can be employed. In transfer learning, information obtained through a pre-trained model using a large dataset (such as ImageNet) is transferred to a smaller dataset. For CNNs, one transfer learning strategy is to modify training in the convolutional layers, such that training occurs only during the last few convolutional layers to perform a prediction. This is based on the premise that the early convolutional layers extract low-level features that can be generalized across images, whereas the later convolutional layers are geared toward identifying high-level features within an image. Low-level features are local and include features such as edges and blobs. High-level features include objects, their states, and events in images, which are extracted using machine-learning techniques. Further strategies include fine-tuning all layers of the CNN by adjusting the weights of the pre-trained network or utilizing a pre-trained model that includes the CNN checkpoints and fine-tuning the network weights. Checkpoints allow pre-trained models to be used for inference without retraining. Alternatively, checkpoints allow model training to resume in case it was interrupted or for the purpose of model fine-tuning.

### Recurrent Neural Network

**Figure 6.**
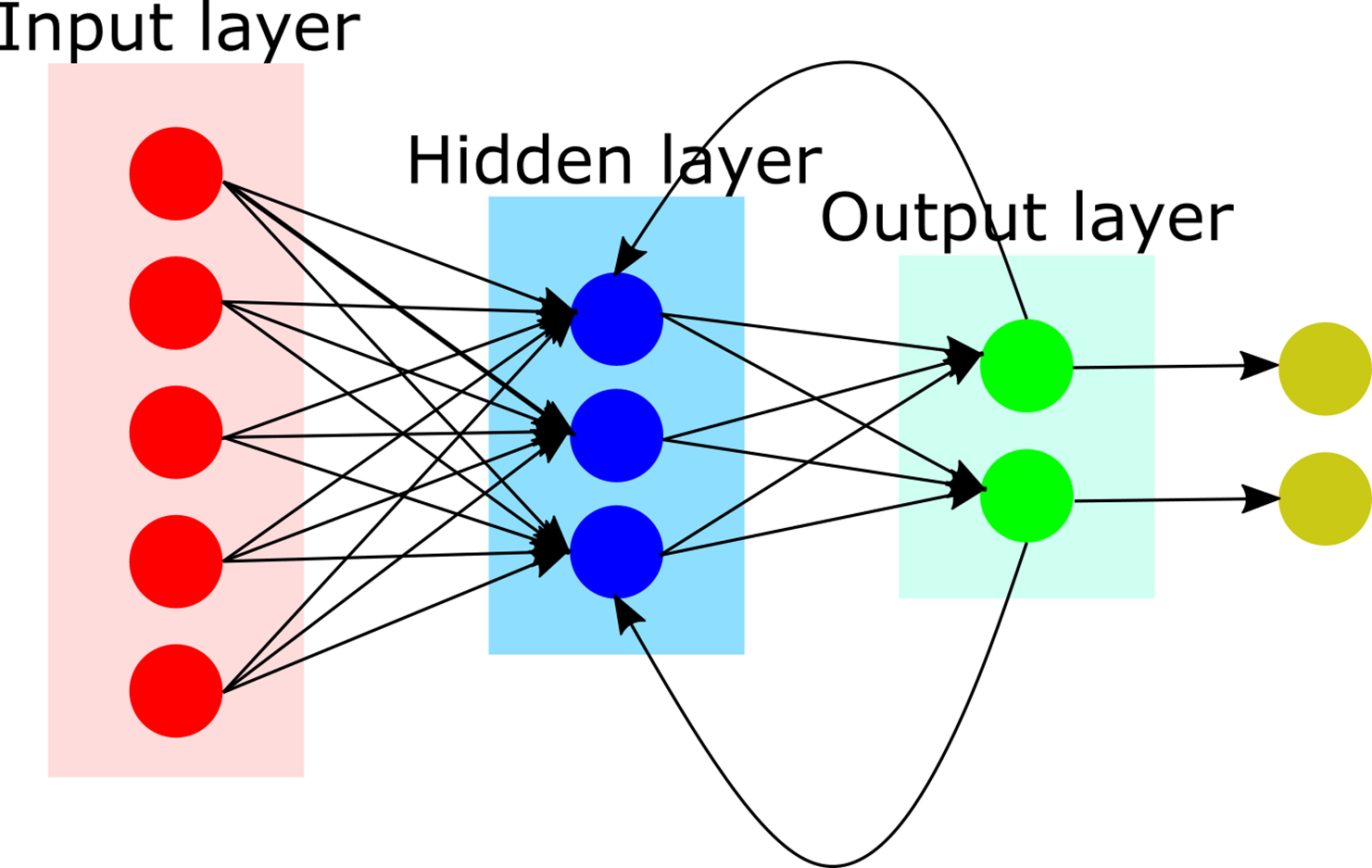
Illustration for the architecture of recurrent neural network (RNN). RNNs are a class of neural network commonly used for text and sequence data. They allow previous outputs to be used outputs to be used as inputs while having hidden states. An important class of RNNs are long-short-term memory (LSTM) which have feedback connections are often used for time series analysis.

Recurrent neural networks (RNNs) are employed to process sequential or time series data, whereby the nodes in RNNs are connected along the data sequence. RNNs are derived from transfer learning FFNNs. While FFNNs allow signals to travel in one direction from input to output only, RNNs allow information to cycle in loops allowing dependencies between data points. Consequently, RNNs possess internal state (memory), where they retain information about past inputs based on its weights and on input data, allowing them to harness past information to predict a later event (Figure 6). RNNs are commonly used for speech and language tasks, such as speech recognition and natural language processing. Of note, long-short-term memory (LSTM) networks are a subtype of RNN that extend the memory of RNNs.

### Autoencoder and deep autoencoder

**Figure 7.**
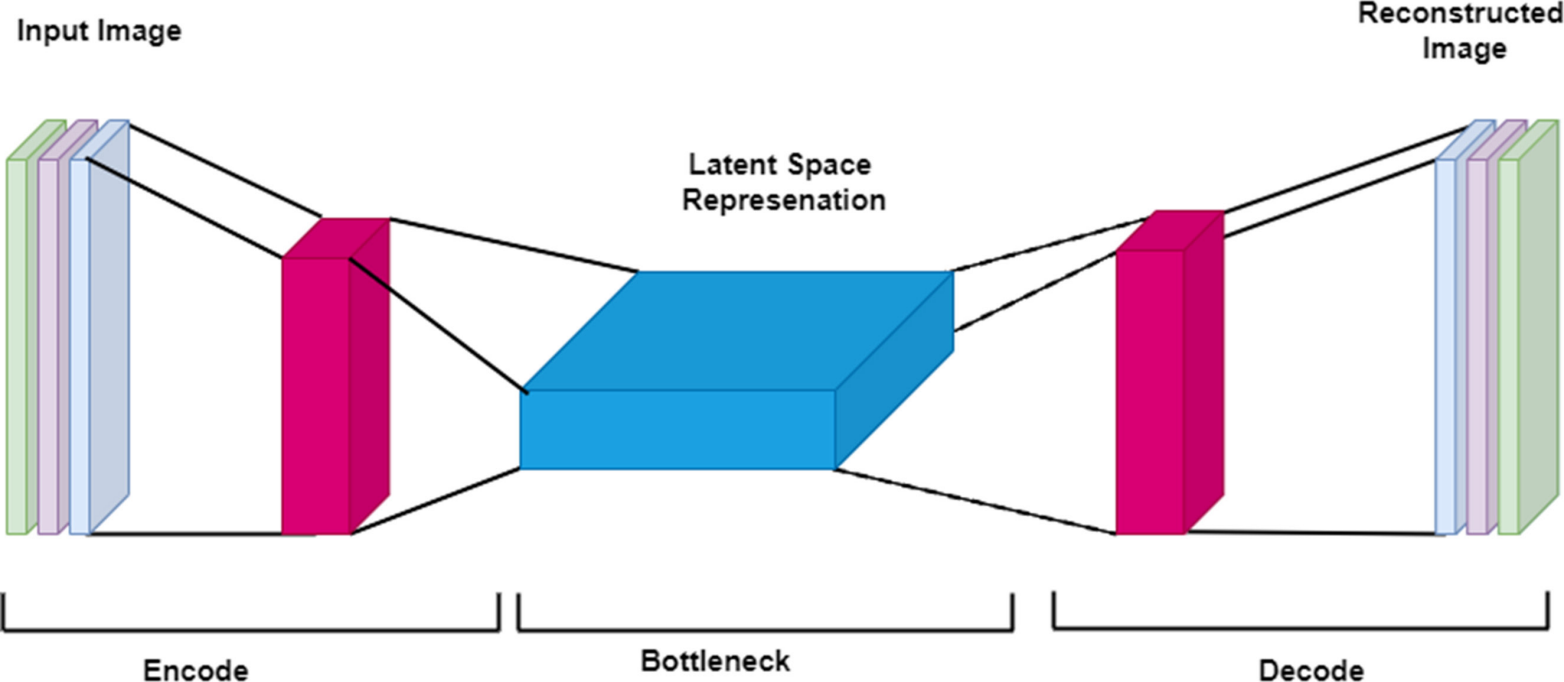
Illustration of a basic autoencoder. An autoencoder is an unsupervised learning model assigned the task of transforming the input image into a latent or compressed representation by minimizing the reconstruction errors between input and reconstructed images of the network. An autoencoder performs two tasks. It first encodes an image, and subsequently it decodes it. Encoding an image in this context means that the autoencoder generates a compressed representation of the original image. Conversely, the decoder takes the output from the bottle neck (latent space representation) and attempts to recreate the input image. For the autoencoder to reconstruct an image, it will need to learn some latent representation of the image. Latent representation refers to a set of compressed features of the image which are learned by the network through an iterative process of training, and which are subsequently used to reconstruct the desired image.

In the 1980s, Geoffrey Hinton designed the autoencoder (AE) to solve unsupervised learning problems. Autoencoders are a type of feedforward neural network for learning representation, in which the network receives the input and deconstructs it into an internal latent representation or code before reconstructing the input as closely to the original image as possible. Autoencoders consist of an input, an output, and multiple hidden layers (Figure 7). The training of the network can be unsupervised, with the goal of reconstruction error minimization: a measure of the differences between the original input and the reconstruction. Types of autoencoder include the multilayer autoencoder; the convolutional autoencoder, intended to reduce image noise or detect video anomalies; and the regularized autoencoder, intended to learn representations for subsequent classification tasks. Regular autoencoders have one layer between the input and output layer, whereas deep autoencoders have multiple hidden layers.

### Generative adversarial network

Generative adversarial networks (GANs) are a branch of DL that I. Goodfellow introduced in 2014.^17^ GANs have been successfully applied to unsupervised image translation, domain adaptation, image in-painting, and semi-supervised classification. They have also been studied for medical image synthesis.^18,19^

**Figure 8.**
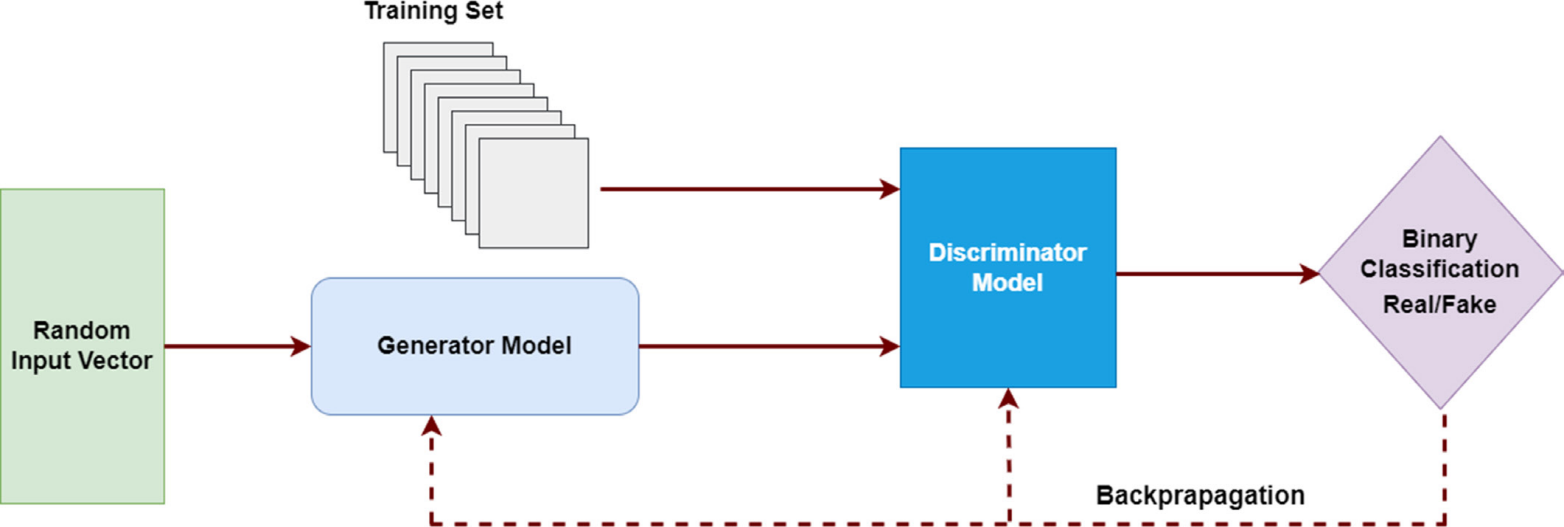
In Generative adversarial networks (GANs) consists of two models: the discriminator and the generator. GANs learn through deriving backpropagation signals through a competitive process involving a pair of networks.

GANs entail simultaneous training of two adversarial models. The GAN architecture is composed of two networks, a Generator (G) and a Discriminator (D), which are trained in competition based on the two-person zero-sum game in game theory (one’s win is another’s loss). The Generator is responsible for generating data, and the Discriminator for estimating the probability that an image was drawn from the training data (is real) or produced by the generator (is fake). The objective of these models is to learn the training data distribution and subsequently generate realistic data samples indistinguishable from the input data. They perform this task by minimizing the loss function through a second adversarial network. During training, the Generator increasingly improves image generation until the Discriminator can no longer distinguish between the real and fake data (Figure 8).

## Treatment response applications

### Brain cancer

Nie et al^20^ used multimodal images of 68 patients with high-grade gliomas to develop a 3D DL framework to predict the survival time (long *vs* short) of patients with high-grade glioma. An independent dataset of 25 patients was used to validate the model. Their approach consisted of a multichannel architecture of 3D CNNs to identify and extract high-level features from *T*_1_-weighted MRI, resting-state functional MRI, and diffusion tensor MRI. Using extracted features as well as demographic and tumor-related features like gender, age at diagnosis, tumor location, tumor size, and WHO grade, a support vector machine was used to predict overall survival time. The combination of deeply learned as well as demographic and tumor-related features resulted in a classification accuracy of 90.66% with threefold cross-validation, and 90.46% with 10-fold cross-validation.

In another study predicting overall survival in patients with glioblastoma multiforme, a proposed radiomics model used deep features extracted from CNNs based on transfer learning and handcrafted features based on radiomics analysis.^21^ The study consisted of 75 patients for training and an independent data set of 37 patients. Both handcrafted features (*N* = 1403) and deep features (*N* = 98304) were extracted from the preoperative multimodality MR images. After feature selection, a model was generated and a radiomics nomogram was constructed by combining signature and clinical risk factors. The radiomics signature outperformed traditional clinical risk factors such as age and the Karnofsky Performance Score for the prediction of overall survival (C-index = 0.710). The model combining the radiomics signature and traditional clinical risk factors further improved prediction performance (C-index = 0.739).

In a study by Kickingereder et al, a DL model using ANNs was developed for the quantitative assessment of tumor response.^22^ Three datasets were used to train and test the model: Heidelberg training dataset (455 patients with brain tumors), Heidelberg test dataset (longitudinal dataset of 40 patients with data from 239 MRI scans), and EORTC-26101 test dataset (MRI scans from 532 patients obtained from 34 institutions). Using the Heidelberg training dataset, an ANN was developed for automated volumetric segmentation of contrast-enhanced tumors and non-enhancing T2-signal abnormalities on MRI. This ANN was derived from the authors’ previously developed ANN, itself based on a U-Net architecture.^15^ The newly developed ANN was asked to predict segmentation masks of contrast-enhanced tumors and non-enhancing abnormalities via an ANN ensemble model (five ANN models obtained from cross-validation of the Heidelberg training dataset) on the Heidelberg and EORTC-26101 test datasets. The tumor segmentation masks generated by the ANNs were shown to be highly accurate in comparison with a reference standard selected as the ground truth segmentation masks generated by a radiologist (median DICE coefficient = 0.89 for contrast-enhanced tumors and 0.93 for non-enhanced abnormalities in the Heidelberg test dataset; 0.91 for contrast-enhanced tumors and 0.94 for non-enhanced abnormalities in the EORTC-26101 test dataset). Moreover, the time to progression determined using ANN-based assessment of tumor response outperformed central RANO assessment for the prediction of overall survival in the EORTC-26101 test dataset (hazard ratios = 2.59 vs. 2.07; *p* < 0.001).

A study by Han et al combined hand-crafted radiomics and deep features generated by a pretrained CNN^23^ from gadolinium-based contrast-enhanced *T*_1_-weighted images of patients with high-grade gliomas from both their institution and from The Cancer Genome Atlas. Feature selection followed by Elastic Net-Cox modeling were performed to predict long- and short-term survivor groups. The model classified patients with high-grade gliomas into long- and short-term survivors (the log-rank test *p* value < 0.001 in patients from their institution, *p* = 0.014 in patients from The Cancer Genome Atlas, and *p* = 0.035 in all patients from both cohorts).

### Breast cancer

In 2012, Hylton et al^24^ reported that MRI outperformed clinical assessment in predicting pathologic complete response (pCR) to neoadjuvant chemotherapy (NAC), using MR images from 216 patients enrolled in the ACRIN 6657/1-SPY1 TRIAL. Tumor measurements on MRI were superior to clinical examination in predicting pCR to NAC at all timepoints. Particularly, tumor volume change at the second MRI examination obtained after one cycle of anthracycline-based treatment showed greatest predictive ability. This work motivated additional studies on the use of MRI to predict pCR, including those with DL tools.

Huynh et al^25^ compared CNN-extracted features from DCE-MR images at different contrast timepoints to determine which timepoint would result in the best classifier for predicting response to NAC, finding that CNN-extracted features based on pre-contrast time points yielded the best classifier.

Several studies investigated deep learning applied to MRI to evaluate response to NAC, using publicly available MR images from the multiinstitutional I-SPY1 TRIAL. Ravichandran et al^26^ applied a CNN to pre-treatment dynamic contrast-enhanced MR images from 166 patients with breast tumors of at least 3 cm in size, who received DCE-MRI imaging prior to treatment, had at least two post-contrast phases of DCE-MRI, and had undergone post-NAC surgery. The classifier to predict pCR based on CNN-extracted features from both pre- and post-contrast images achieved an accuracy of 82% in the testing set. The inclusion of HER2 status to the classifier improved the accuracy to 85%. Another study using MR images from the I-SPY TRIAL, Liu et al,^27^ developed a CNN algorithm to predict pCR *vs* no-pCR response to NAC based on post-contrast images only, which yielded an accuracy of 72.5%. Due to the high computational burden associated with training customized CNNs, Comes et al^28^ investigated a transfer learning approach, using the pre-trained CNN AlexNET (previously trained to extract both low-level features such as edge and dots and high-level features such as shapes and objects from a raw image), to evaluate the early efficacy of NAC before the completion of therapy. When optimized features extracted from pre- and early treatment exams were combined with clinical features such as ER, PgR, HER2 and molecular subtypes, the classifier achieved an accuracy of 91.4% on the subset of patients used for fine-tuning, and 92.3% on the independent database.

Single-institution studies have also shown that deep learning is promising to predict response to NAC. Ha et al^29^ investigated a CNN to predict NAC response based on pre-treatment breast MRI for 141 patients with locally advanced breast cancer who had pre-treatment MRI followed by adriamycin/taxane-based NAC and surgical resection. Patients were divided into three groups based on NAC response: complete, partial, and no response/progression. Tumors underwent 3D segmentation on the first post-contrast image. The CNN architecture consisted of ten convolutional layers, four max-pooling layers, and 50% dropout after a fully connected layer. The overall mean accuracy of the CNN was 88% (95% CI,±0.6%). In another study, the same group of authors^30^ developed a CNN algorithm to predict post-NAC pCR of the axilla using breast MRI performed before NAC. The proposed CNN algorithm achieved an overall accuracy of 83%. El Adoui et al^31^ evaluated a group of 42 breast cancer patients who had DCE-MR imaging before and after the first cycle of chemotherapy and developed a CNN that achieved an area under the receiver operating characteristic curve (AUC) of 0.91 and accuracy of 88% using both the pre- and post-treatment examinations without segmentation (multi-input CNN). Using single-input CNN of pre-treatment examinations only or post-treatment examinations only with or without segmentation achieved an AUC of 0.69–0.79 and accuracy of 68–80%.

In applying DL using both positron emission tomography/magnetic resonance imaging (PET/MRI) scans obtained before and after the first cycle of NAC in patients with advanced breast cancer, Choi et al^32^ generated CNNs based on AlexNet that improved the classification of patients into pCR and non-pCR groups compared with the majority of conventional PET and MR imaging parameters.

### Colorectal cancer metastases

Colorectal liver metastases (CRLM) are the third leading cause of cancer-related death in the US.^33^ The assessment of treatment response at preoperative chemotherapy is crucial to inform therapeutic adjustments that maximize benefit. Zhu et al^34^ applied DL to MR images to predict CRLM response to chemotherapy. The study included 101 patients in the training cohort, 54 patients in the testing cohort, and an additional 25 patients as an external validation cohort. The DL architecture was designed to import four inputs: pre- and post-treatment *T*_2_-weighted image, and pre- and post-treatment apparent diffusion coefficient (ADC) images. The network was designed to extract features from the input data to distinguish pathology tumor regression grade (TRG) between the response and non-response group, as well as to distinguish survival outcomes after hepatectomy. Three models were developed: Model A (based on pre- and post-treatment MRI), Model B (based on pre-treatment MRI only), and Model C (based on post-treatment MRI only). The results of the DL algorithm were compared with RECIST to predict tumor response and determine survival outcome. The accuracy of Model A (accuracy of 87.5%) was significantly higher as compared with Models B and C (accuracy of 79.7 and 85.9%, respectively) and RECIST (accuracy of 57.8%). The *p*-values for comparison were as follows: 0.04 for comparison of Model A *vs* Model B, 0.04 for comparison of Model A *vs* Model C, and 0.03 for comparison of Model A *vs* RECIST.

### Rectal cancer

The current standard-of-care treatment in patients with locally advanced rectal cancer (LARC) is neoadjuvant chemoradiation therapy (CRT) followed by total mesorectal excision (TME). Patients with pCR may be spared resection if followed with biopsy and MRI.^35^ Assessment of response to chemoradiotherapy can impact treatment decision-making for these patients. The availability of additional treatment options or non-operative approaches is a motivating factor for the assessment of treatment response. MRI plays an important role in treatment response assessment after chemoradiotherapy. However, distinguishing between therapy-induced scarring and residual viable tumor on *T*_2_-weighted sequences remains difficult.^36^

Recently, radiomics and DL methods have been used to predict pCR in patients with LARC. In a study, Shi et al^37^ extracted radiomic features from pre-treatment MRI T1- and *T*_2_-weighted images, axial DWI, and *T*_1_-weighted DCE-MRI. They used a three-layer ANN to select parameters and build diagnostic radiomics models. Additionally, a CNN was developed with the image input a tight bounding box covering the tumor region of interest (ROI). Results showed that CNN based on pre-treatment and mid-radiation therapy MRI achieved an AUC of 0.83 for predicting pCR *vs* non-pCR, whereas the model combining ROI and radiomic features achieved an AUC of 0.80 based on pre-treatment images, 0.82 for mid-radiation therapy, and 0.86 for both pre-treatment and mid-radiation therapy images.

Radiomics methods provide a valuable mechanism for extraction of quantitative features from medical images. These can then be correlated with various biological features and clinical endpoints. Delta-radiomics is an emerging approach and an extension of radiomics based on the analysis of variations of radiomics features at different acquisition time points.^38^ The points are typically pre- and post-treatment, with the objective of predicting response.^39^ Delta-radiomic features have shown promise in predicting the response of colorectal liver cancer^40^ and metastatic renal cell cancer^41^ to chemotherapies, as well as the analysis of CT images to determine the treatment response of non-small cell lung cancer to radiation therapy.^42^ One study, evaluating the ability of delta-radiomics to predict overall survival of patients with recurrent malignant gliomas who were treated with concurrent stereotactic radiosurgery and bevacizumab, indicated that delta-radiomic features potentially provided better treatment assessment than features extracted from a single time point.^43^ While delta-radiomics is at an early stage, it has shown promising results in studies focusing on temporal changes of radiomic features in treatment response assessment. Delta-radiomics provides high-dimensional data, making machine-learning tools like DL suitable for feature analysis. An in-depth review providing detailed information on delta-radiomics is available elsewhere.^38^

In another study, Zhang et al^44^ developed DL models to predict response based on diffusion kurtosis and *T*_2_-weighted MRI from 383 patients with LARC who underwent baseline MRI prior to preoperative chemotherapy. The DL network architecture consisted of a multipath CNN with eight inputs comprising *T*_2_-weighted imaging and diffusion kurtosis imaging pre- and post-treatment.^45^ Three DL models were considered. The first was for pCR prediction and second for TRG (0 + 1) and TRG (2 + 3) classification. The third model was for T-downstage and non-T-downstage classification. The first model for pCR prediction achieved an AUC of 0.99, significantly better than the evaluation by two radiologists (AUC of 0.66 for rater 1 and 0.72 for rater 2) (*p* < 0.001). The second and third models had AUC of 0.70 and 0.79, respectively. The DL model also served to reduce radiologist error rate; when radiologists were assisted by the DL model in predicting pCR, their AUC significantly improved to 0.82 for rater 1 and 0.83 for rater 2 (*p* = 0.002 and 0.01, respectively). However, the diagnostic performance of the DL models for classifying TRG and T stage downgrading did not exceed the two radiologist evaluations.

As compared to radiomics analysis, applying DL methodology to evaluate tumor response to treatment using MRI offer several key advantages. First, DL approaches typically do not require precise tumor delineation. Second, they often outperform radiomic feature analysis. Third, they automatically learn and hierarchically organize task-adaptive image features. The extracted features might not be visually identifiable but reflect associations between the classifier and images, providing tremendous potential in clinical decision-making.

### Challenges and opportunities of Deep-learning

DL has both numerous advantages over traditional machine learning and tremendous potential to transform MRI-based evaluation of tumor treatment response. CNN, a popular DL architecture, allows the network to independently learn by performing prediction tasks, such as identification of useful regions or extraction of salient features from those regions, without the need for human intervention.^46,47^ CNN provides a general-purpose learning procedure for an end-to-end image analysis workflow. CNNs learn specific patterns of their given task from the images themselves instead of relying on preprocessing steps, ‘handcrafted’ features, or subsequent model building. The objective is for the network to automatically extract relevant features from images, resulting in easy clinical application. However, some challenges warrant consideration when constructing a network that incorporates DL into clinical decision-making. We present these below.

### Data availability and annotation

A key challenge is the availability of data, specifically medical images related to the clinical task at hand. The lack of sufficient data for training DL models in medical image analysis can limit the ability of deep neural networks to perform adequately. This problem is further exasperated by the time-consuming, expensive, and error-prone process of medical imaging annotation. One common solution is to transfer learning from pretrained models, *e.g*., ImageNet. However, this approach could be ineffective in many instances due to differences in learned features between natural and medical images. To overcome the challenges associated with transfer learning, several novel approaches have been proposed.^48–50^ Alzubaidi et al proposed training the DL model on large, unlabeled medical image datasets. This knowledge is then transferred to train the DL model on the small amount of labeled medical images.^50^

### Overfitting and class imbalance

Given the large number of parameters that need to be optimized, a major concern in DL is insufficient disease representation, which may result in overfitting or class imbalance. The design and evaluation of DL networks should consider the risks associated with overtraining and overfitting of a particular network, which can lead to poor performance on data that has not been used for training purposes. A reliable network must incorporate sufficient instances of disease and/or rare diseases that might not be fully reflected within the network architecture and could therefore lead to reduced performance.^51^ Several solutions have been proposed to address the class imbalance problem. These include training the network with random undersampling, or removing some observations of the majority class; random oversampling, or higher sampling of the minority classes; Synthetic Minority Oversampling Technique (SMOTE)^52^ ; the NearMiss family of methods,^53^ which is an undersampling technique; and penalizing learning algorithms, which is a cost-sensitive training; among others.

### Data bias

At least seven types of data bias have been identified in machine learning literature, including sample bias, exclusion bias, measurement bias, recall bias, racial bias, and association bias. A complete survey of bias and fairness in machine learning is beyond the scope of this paper but the reader is referred to^54^ for further details. Tools exist to address these issues, such as dividing datasets to train the model and including datasets from multiple testing centers. Further data availability and the sharing of MR images across institutions would mitigate concerns regarding generalizability, as well as enhance confidence in the reliability of DL methods for clinical use.

### Interpretability: the ‘black-box’ approach

Another important challenge associated with DL models is the ‘black-box’ approach, which focuses primarily on optimizing outcome performance. It provides limited insight into internal structures or features of the models that lead to treatment decisions based on given model inputs. This limitation effectively diminishes the confidence required for such implementations to be broadly accepted within a clinical setting. To address this well-recognized challenge, several investigators have advocated for approaches based on interpretable models from the beginning^55^ 155. At present, there is no consensus on the proposed approach. Divergence exists among researchers who highlight interpretability of the models.^55^ For example, while Alex John London advocates optimal performance and predictive power as the primary basis for model evaluation,^56^ others prefer models that are highly transparent. They refer to these as ‘explainable medicine’ and require causality.^57^ This is an active area of research that will have a significant impact on the trajectory of the field.

### Regulatory approval, ethical challenges, and reimbursement

Several key obstacles need to be overcome for DL methods to be widely accepted in a clinical setting. These include regulatory approval, which requires FDA approval in the USA and a separate approval process within the European Union. Further, DL implementation in clinical practice requires that legal and ethical issues of liability be resolved ahead of time. Finally, there must be a mechanism in which radiology AI can be reimbursed for usage. The current state of affairs is carefully reviewed by Chen et al..^58^
